# Characterization of a New *Pm2* Allele Conferring Powdery Mildew Resistance in the Wheat Germplasm Line FG-1

**DOI:** 10.3389/fpls.2016.00546

**Published:** 2016-04-26

**Authors:** Pengtao Ma, Hongxng Xu, Lihui Li, Hongxia Zhang, Guohao Han, Yunfeng Xu, Xiaoyi Fu, Xiaotian Zhang, Diaoguo An

**Affiliations:** ^1^Center for Agricultural Resources Research, Institute of Genetics and Developmental Biology – Chinese Academy of SciencesShijiazhuang, China; ^2^The National Key Facility for Crop Gene Resources and Genetic Improvement, Institute of Crop Science – Chinese Academy of Agricultural SciencesBeijing, China; ^3^Shijiazhuang Academy of Agricultural and Forestry SciencesShijiazhuang, China

**Keywords:** allelic variation, *Blumeria graminis*, MAS, *Triticum aestivum*, *Pm2* locus

## Abstract

Powdery mildew has a negative impact on wheat production. Novel host resistance increases the diversity of resistance genes and helps to control the disease. In this study, wheat line FG-1 imported from France showed a high level of powdery mildew resistance at both the seedling and adult stages. An F_2_ population and F_2:3_ families from the cross FG-1 × Mingxian 169 both fit Mendelian ratios for a single dominant resistance gene when tested against multiple avirulent *Blumeria tritici* f. sp. *tritici* (*Bgt*) races. This gene was temporarily designated *PmFG*. *PmFG* was mapped on the multi-allelic *Pm2* locus of chromosome 5DS using seven SSR, 10 single nucleotide polymorphism (SNP)-derived and two SCAR markers with the flanking markers *Xbwm21*/*Xcfd81*/*Xscar112* (distal) and *Xbwm25* (proximal) at 0.3 and 0.5 cM being the closest. Marker *SCAR203* co-segregated with *PmFG*. Allelism tests between *PmFG* and documented *Pm2* alleles confirmed that *PmFG* was allelic with *Pm2*. Line FG-1 produced a significantly different reaction pattern compared to other lines with genes at or near *Pm2* when tested against 49 *Bgt* isolates. The *PmFG*-linked marker alleles detected by the SNP-derived markers revealed significant variation between FG-1 and other lines with genes at or near *Pm2*. It was concluded that *PmFG* is a new allele at the *Pm2* locus. Data from seven closely linked markers tested on 31 wheat cultivars indicated opportunities for marker-assisted pyramiding of this gene with other genes for powdery mildew resistance and additional traits.

## Introduction

Powdery mildew of wheat, caused by *Blumeria graminis* f. sp. *tritici* (*Bgt*), is a foliar disease that occurs worldwide, especially in wheat-producing regions with maritime or semi-continental climates (Cowger et al., 2012). In China, wheat yields and quality have been affected by this disease since the 1970s, especially high-yielding cultivars grown with high-inputs of irrigation and fertilization (Bennett, 1984; Sun et al., 2015). Over the past decade, the area affected by powdery mildew in China has ranged from 6 to 8 million hectares each year, resulting in estimated grain losses of 300,000 metric tons^1^.

Although, fungicides can reduce losses in yield and quality caused by powdery mildew, resistant cultivars are the preferred means for control (Horst, 2013; Wang et al., 2015b). More than 70 formally named and about 20 temporarily designated powdery mildew (*Pm*) resistance genes/alleles have been identified (McIntosh et al., 2013; Hao et al., 2015). However, due to the race-specific nature of resistance and excessive deployment of single resistance genes, the effectiveness of *Pm* genes is often short-lived as they are defeated by virulent mutants of the pathogen (Hsam and Zeller, 2002; Xiao et al., 2013). Detailed studies have indicated that most current wheat cultivars grown in China have non-effective or no *Pm* genes (Li et al., 2011). It is therefore urgent to identify effective sources of resistance among germplasm from around the world to increase the existing genetic diversity.

Some of the documented *Pm* loci have multiple resistance alleles, such as *Pm1* (Hsam et al., 1998; Singrün et al., 2003), *Pm2* (Ma et al., 2015a; Xu et al., 2015), *Pm3* (Zeller and Hsam, 1998), *Pm4* (Schmolke et al., 2012), *Pm5* (Hsam et al., 2001; Huang et al., 2003), and *Pm24* (Huang et al., 2000; Xue et al., 2012). Although, some alleles at these loci may have lost their effectiveness, further allelic variation may be present in other germplasm. The gene *Pm2* was identified several decades ago (Pugsley and Carter, 1953), and was used as an effective resistance source in some countries (Li et al., 2011). Although, the avirulence frequency remains low in some regions of China and other parts of the world after decades of deployment, several new alleles (e.g., *Pm2b*, *Pm2c*, *PmLX66*, and *PmW14*) were identified, thereby increasing the diversity of available resistance genes (Ma et al., 2015a; Sun et al., 2015; Xu et al., 2015).

Molecular markers are powerful tools for tagging resistance genes. Almost all the designated *Pm* genes have been mapped to specific chromosomal loci (McIntosh et al., 2013). Microsatellites or simple sequence repeats (SSRs) provide a simple and effective marker system for molecular mapping in wheat (Somers et al., 2004; Sourdille et al., 2004; Xue et al., 2008). To develop high-density marker assays, high-throughput single nucleotide polymorphism (SNP) genotyping platforms based on wheat 9K, 90K, and even 660K SNP chips are now available (Bérard et al., 2009; Lai et al., 2012; Avni et al., 2014; Wang et al., 2014), and these will greatly increase the numbers of markers closely linked to targeted resistance genes.

Molecular markers closely linked to targeted genes controlling valuable traits can be used to rapidly transfer them to other cultivars. Hence, marker-assisted selection (MAS) has been practiced in many parts of the world (USA, Australia, Canada, India, and Europe) to complement conventional breeding programs (Gupta et al., 2010). A number of markers associated with documented QTL/genes for some major economic traits, such as disease resistance, grain protein content and pre-harvest sprouting tolerance, have also been used for MAS in wheat breeding programs (e.g., de Bustos et al., 2001; Davies et al., 2006; Nocente et al., 2007; Badea et al., 2008; Zhang et al., 2009).

In this study, the wheat germplasm line FG-1 imported from France showed a high level of powdery mildew resistance in China. To make better use of this resistance resource, the following research was carried out to: (1) determine the inheritance of powdery mildew resistance in FG-1 using an array of *Bgt* isolates, (2) determine the chromosomal location of the resistance gene using different kinds of molecular markers and allelism tests, (3) compare response spectra of FG-1 and lines carrying documented *Pm* genes using *Bgt* isolates, (4) to compare allelic variation between FG-1 and genotypes with documented *Pm* genes using SNP-derived markers, and (5) investigate the applicability of closely linked markers for MAS.

## Materials and Methods

### Plant Materials

FG-1 is a common wheat line that was imported from France and maintained in the germplasm bank of Shijiazhuang Academy of Agricultural and Forestry Sciences (Shijiazhuang, Hebei Province, China) of unknown pedigree. It has been grown in Northern China since it was imported from France. It has resistance to powdery mildew at both the seedling and adult growth stages based on observations over many years. The susceptible Chinese cultivar (cv.) Mingxian 169 was crossed with FG-1 to study the inheritance of powdery mildew resistance. Mingxian 169 and Huixianhong were used as susceptible controls for test of powdery mildew resistance. Several stocks with documented *Pm* genes on chromosome arm 5DS, such as Ulka/8*Cc with *Pm2a*, KM2939 with *Pm2b*, Niaomai with *Pm2c*, Tabasco with *Pm48*, Liangxing 66 with *PmLX66*, Wennong 14 with *PmW14*, YingBo700 with *PmYB*, Zhongmai 155 with *PmZ155*, X3986-2 with *PmX3986-2*, Wanfengjian 34 with *PmWFJ*, PB3558 with *PmPB3558*, Brock with *MlBrock* and D57-5D with *PmD57-5D* were used in multi-race response comparisons with FG-1. Thirty-one wheat cultivars representing Chinese elite germplasm were tested using molecular markers closely linked to the *Pm* gene in FG-1 to validate their applicability for MAS.

### Phenotyping Reactions to Powdery Mildew

Forty-nine *Bgt* isolates with different avirulence/virulence arrays (races) collected from different regions of China (Supplementary Table S1) were used to inoculate FG-1 and various host lines to determine the breadth of effectiveness of the resistance in FG-1 and compare the response spectrum of FG-1 to wheat stocks with documented *Pm* genes. Five seedlings per plot were planted in rectangular trays with 128 cells (3 cm × 3 cm) in a growth chamber. The susceptible check Mingxian 169 and Huixianhong were randomly planted in the trays. Three replications were included in each test. When the seedlings reached the one-to two-leaf stage, fresh conidiospores from Mingxian 169 seedlings were dusted on the trays. The trays were then placed in a greenhouse with a daily cycle of 14 h light at 22 ± 2°C and 10 h of darkness at 18 ± 2°C. The inoculation operations were performed once a day for three consecutive days. When the disease was fully developed on Mingxian 169, the infection types (ITs) on each plant were assessed on a 0–4 scale based on the IT scale described by Si et al. (1992), with ITs 0, 0, 1, and 2 being regarded as resistant, and ITs 3 and 4 as susceptible. All the phenotypic experiments were repeated three times to confirm their reactions to each *Bgt* isolate.

The adult plant reactions to powdery mildew of FG-1 and the susceptible controls Mingxian 169 and Huixianhong were evaluated under field conditions using a mixture of the *Bgt* isolates prevalent in northern China. The tests with the mixture of the isolates were conducted using the procedures described by An et al. (2013) at Luancheng Agro-Ecological Experimental Station, Chinese Academy of Sciences, Shijiazhuang, China. Disease reactions were assessed on a 0–9 scale, where 0–4 was considered resistant and 5–9 susceptible (An et al., 2013). The adult plant reactions test was repeated over 4 years’ growing season using the same procedure.

*Bgt* race B03 was chosen to inoculate seedlings of the segregating materials and parents for genetic analysis. Twenty-four plants of each F_2:3_ family were inoculated with this race. To confirm that the same resistance gene conferred the resistance to all avirulent *Bgt* isolates, random sets of 10 homozygous resistant and 10 segregating F_2:3_ families to the isolate B03 were tested with all of the 42 *Bgt* isolates that were avirulent on FG-1. Twenty-four plants of each F_2:3_ family were inoculated with each tested race. The number of resistant and susceptible plants was counted to confirm the phenotype of each F_2:3_ family. Tests on F_2:3_ families with intermediate ITs 2 and 3 were repeated to confirm the results of the previous tests for each *Bgt* isolate.

### Molecular Genotyping of the Mapping Population

Genomic DNA of FG-1, Mingxian 169 and the F_2_ plants were isolated using the phenol/chloroform method (Sharp et al., 1988) following evaluation of their powdery mildew reactions. Resistant and susceptible DNA bulks were produced using equal amounts of DNA from 10 homozygous resistant and 10 homozygous susceptible F_2_ plants, respectively, following progeny testing. To screen for molecular markers potentially linked to the *Pm* gene in FG-1, the parents FG-1 and Mingxian 169 and resistant and susceptible bulks were assessed for polymorphisms, initially using 50 SSR markers linked to 35 documented *Pm* genes. To saturate the linkage map, different types of molecular markers were screened further. They included about 100 SSR markers on chromosomes arms 4DL, 5DS, and 7BS from Somers et al. (2004), 25 SNP-derived markers located on chromosome 5DS developed from the wheat 90K SNP array and its sequence alignment with the *Aegilops tauschii* draft genome sequence (Lu et al., 2015), 25 EST-derived markers located on chromosome arm 5DS and two sequence characterized amplified region (SCAR) markers linked to documented *Pm2* alleles (Li et al., 2009; Huang et al., 2012). Polymorphic markers were then genotyped on the F_2_ population of FG-1 × Mingxian 169. After confirming the response genotypes through progeny testing of F_2:3_ families, a linkage map for the resistance gene in FG-1 was then produced using the software MAPMAKER 3.0 with a LOD threshold of 3.0 and a maximum map distance 37.5 cM (Lincoln et al., 1993). Kosambi function was carried out to calculate the map distances between linked markers and the targeted gene using a total of 214 F_2:3_ families from the cross FG-1/Mingxian 169 (Kosambi, 1943). Chi-squared tests were performed to determine the goodness of fit of observed and expected segregation ratios of the F_2_ and F_2:3_ populations for markers and powdery mildew responses.

### Allelism Test

After the *Pm* gene(s) in FG-1 was mapped on the short arm of chromosome 5D, FG-1 was crossed with the resistant stocks with documented *Pm* genes on the same chromosome arm to obtain F_2_ populations. The *Bgt* isolate B03, which was avirulent to FG-1 and all the documented resistant stocks, was selected to inoculate the F_2_ populations. The susceptible check Mingxian 169 and Huixianhong were randomly planted in the trays. The number of resistant and susceptible plants in each plot was counted after evaluating their phenotyping reactions. Then, the allelic relationships between the *Pm* gene(s) in FG-1 and documented *Pm* genes on the same chromosome arm were confirmed based on the ratio of resistant and susceptible F_2_ plants.

### Allelic Variation of Linked Marker Alleles

To compare the *Pm* gene(s) in FG-1 and documented allelic and closely linked *Pm* genes, several markers that were linked with *PmFG* were selected to amplify FG-1 and resistant stocks with *Pm* genes at or near *Pm2* locus. The allelic sizes of those markers were assessed by the SensiAnsys gel imaging analysis system (Shanghai Peiqing Science & Technology Ltd., Shanghai City, China).

### Validation of the Closely Linked Markers in Different Genetic Backgrounds

To evaluate the potential of the *Pm* gene(s) in FG-1 for MAS, several closely linked markers were assayed in 31 Chinese wheat cultivars. The patterns or sizes of the polymorphic bands amplified from these cultivars were compared with those amplified from FG-1 to assess the usefulness of the markers in MAS. If the polymorphic band(s) of a marker were all same for FG-1 and the wheat cultivars, it could not be used for MAS. However, patterns or sizes of the polymorphic bands amplified from FG-1 that differed from those in the wheat cultivars indicate the marker can be used to detect *PmFG* when it was transferred into those cultivars by hybridization.

## Results

### Evaluation and Inheritance of Powdery Mildew Resistance in FG-1

**FIGURE 1:**
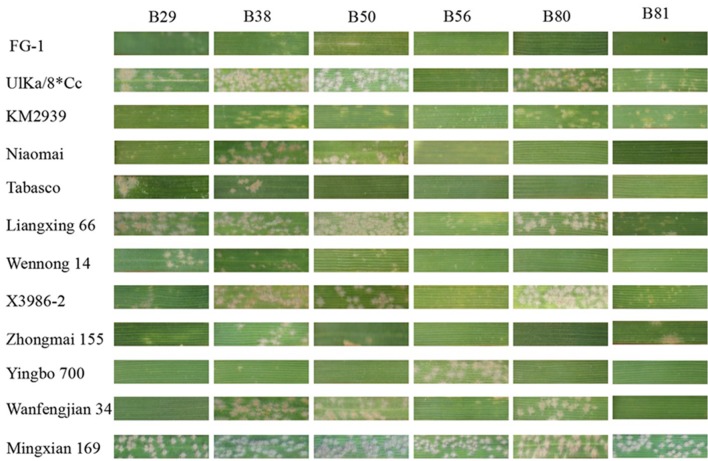
**Examples of leaf segment reactions of FG-1 and various wheat genotypes to 6 of 49 *Bgt* isolates; cv. Mingxian 169 was used as the susceptible control**.

FG-1 showed resistance to a mixture of *Bgt* races in the field over 4 years with disease reaction types 0-2 while adjacent controls Huixianhong and Mingxian 169 were susceptible with disease reaction types 8–9. At the seedling stage, FG-1 was resistant to 43 of the 49 *Bgt* isolates collected from different regions, indicating an avirulence frequency of 87.8% (**Figure 1**; Supplementary Table S1). Compared with several wheat cultivars currently deployed in different regions of China, FG-1 possessed a broader resistance spectrum, and more significantly, it was resistant to several highly virulent *Bgt* isolates; for example, B29, B38, B50 and B80, which are virulent on several or all of the five cultivars Liangxing 66, Liangxing 99, Wennong 14, Zhongmai 155, and Jimai 22 widely planted in Shandong, Hebei, and Henan provinces and Beijing area. Therefore, FG-1 could serve as a valuable resistance donor to add to the current diversity of *Pm* genes in different wheat production regions.

When tested with *Bgt* isolate B03, FG-1 was resistant with an IT 0, while the cultivar Mingxian 169 was susceptible with an IT 4. The infection types of the F_1_s, and the segregation patterns of the F_2_, and F_2:3_ populations are shown in **Table 1**. F_1_ plants from the cross showed infection types similar to the resistant parent, indicating that the resistance was dominant. We observed segregation of 228 resistant: 74 susceptible in the F_2_ population, which is consistent with an expected segregations for a single dominant locus. The F_2_ population was then transplanted to the field and 214 plants produced enough seed for progeny testing. When tested with the same race, segregations of 54 homozygous resistant (RR), 115 segregating (Rr) and 45 homozygous susceptible (rr) F_2:3_ families confirmed single gene segregation. The gene was tentatively designated *PmFG*.

**Table 1:** Segregation ratios for the powdery mildew reactions of F_1_ plants, and F_2_ and F_2:3_ populations from the cross FG-1 × Mingxian 169 when inoculated with *Bgt* isolate B03 in the greenhouse.

Cross	Plants observed			Expected ratio	χ^2^	*P*
	HR	Seg	HS			
FG-1 × Mingxian 169 F_1_	20	–	0	–	–	–
FG-1 × Mingxian 169 F_2_	228	–	74	3:1	0.02	0.89
FG-1 × Mingxian 169 F_2:3_	54	115	45	1:2:1	1.95	0.37

*Table values of χ^2^ for significance at *P* = 0.05 are 3.84 (1 *df*), 5.99 (2 *df*).*

When tested against 42 other *Bgt* isolates that were avirulent to FG-1, all the 10 homozygous resistant F_2:3_ families for B03 were also homozygous resistant to all avirulent isolates and all the 10 segregating F_2:3_ families for B03 again segregated, including isolates B29 and B38 with IT 2 on FG-1. Therefore, *PmFG* conferred powdery mildew resistance to all the avirulent *Bgt* isolates.

### Molecular Mapping of *PmFG*

**FIGURE 2:**
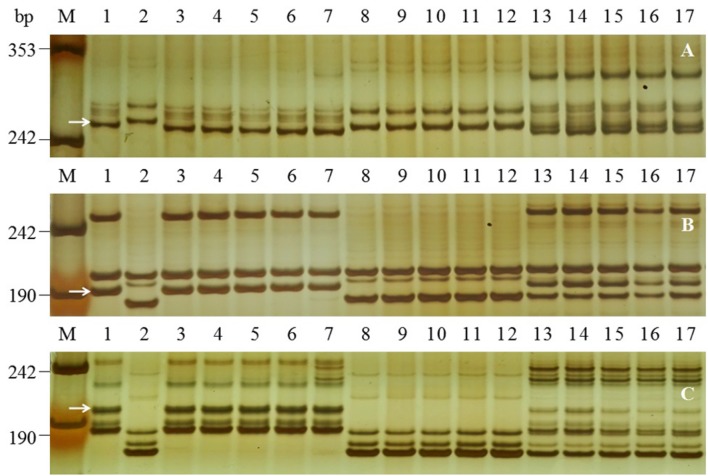
**Examples of amplification patterns of *PmFG*-linked polymorphic SSR marker *Cfd81***(A)** and SNP-derived markers *Bwm20***(B)** and *Bwm25***(C)** in the parents and selected F_2:3_ families of FG-1 × Mingxian 169 in 8% silver-stained non-denaturing polyacrylamide gels.** Lanes M, pUC18 *Msp* I; lanes 1-2: FG-1 and Mingxian 169; lanes 3–7: homozygous resistant F_2:3_ families; lanes 8–12, homozygous susceptible F_2:3_ families; lanes 13–17 heterozygous F_2:3_ families. White arrows indicate the polymorphic band of FG-1.

**FIGURE 3:**
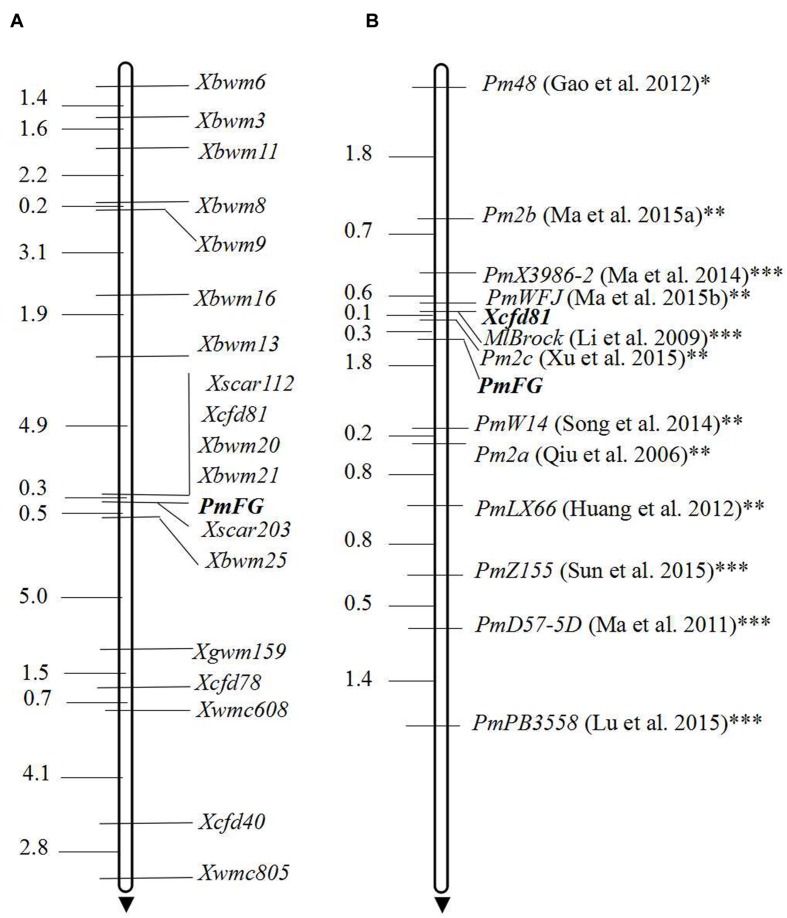
**Linkage map of *PmFG* using the F_2:3_ families of FG-1 × Mingxian 169 **(A)** and its locus comparison with the documented *Pm* genes on chromosome arm 5DS using *PmFG* as point zero with the genetic distances marked off from it based on the anchoring marker locus *Xcfd81***(B)**.** Genetic distances in cM are showed to the left. *, closely linked with *Pm2*; **, allelic with *Pm2*; ***, allelic relationship with *Pm2* was not confirmed.

The *Pm2*-linked SSR marker *Cfd81* was polymorphic for the parents and the bulks. *Cfd81* was genotyped on the the F_2:3_ mapping population (**Figure 2**). Its linkage with *PmFG* was estimated at 0.3 cM (**Figure 3**). Because *Cfd81* also can be amplified at loci on chromosome arms 4DL, 5DS and 7BS, 79 SSR markers located on these chromosome arms (Somers et al., 2004) were screened to confirm the location of locus *PmFG* and to increase the density of the linked markers. SSR markers *Cfd40*, *Cfd78*, *Gwm159*, *Wmc608*, and *Wmc805* on chromosome arms 5DS from the map of Somers et al. (2004) were shown polymorphic and linked to *Pm FG* (**Table 2**; **Figure 3**). Markers reportedly located on chromosome arms 4DL and 7BS (Somers et al., 2004) showed no polymorphisms between the parents and the bulks, indicating that *PmFG* was not located on those chromosome arms. To saturate the linkage map of the *PmFG* region, 10 SNP-derived markers on chromosome arm 5DS and the SCAR marker *SCAR112* which is linked to a documented *Pm2* allele were shown polymorphic and linked to *PmFG* at genetic distances ranging from 0.3 to 16.9 cM (**Table 2**; **Figure 3**). SCAR marker *SCAR203* co-segregated with *PmFG* (**Figure 3**). The reported locations of some of the SNP markers and the SCAR markers were in the deletion bin 5DS1-0-0.63 (Sourdille et al., 2004; Li et al., 2009; Lu et al., 2015), therefore, *PmFG* should also be located in this chromosome bin.

**Table 2:** Characteristics and sizes of the homozygous allele of the FG-1 (AA), homozygous allele of the Mingxian 169 (aa) and heterozygous allele (Aa) of the *PmFG*-linked markers from the reactions of F_2:3_ families of FG-1 and Mingxian 169.

Linked markers	Marker characteristics	Size of AA allele(s) (bp)	Size of aa allele(s) (bp)	Size of Aa allele(s) (bp)
*Bwm3*	SNP-derived, co-dominant	144	147	144, 147
*Bwm6*	SNP-derived, co-dominant	169	167	167, 169
*Bwm8*	SNP-derived, co-dominant	210	204	204, 210
*Bwm9*	SNP-derived, co-dominant	208	203	203, 208
*Bwm11*	SNP-derived, co-dominant	225	227	225, 227
*Bwm13*	SNP-derived, co-dominant	194	190	190, 194
*Bwm16*	SNP-derived, co-dominant	192	190	190, 192
*Bwm20*	SNP-derived, co-dominant	195	182	182, 195
*Bwm21*	SNP-derived, co-dominant	220	260	220, 260
*Bwm25*	SNP-derived, co-dominant	208	156	156, 208
*Cfd40*	SSR, co-dominant	220	214	214, 220
*Cfd78*	SSR, co-dominant	310	312	310, 312
*Cfd81*	SSR, co-dominant	270	275	270, 275
*Wmc608*	SSR, co-dominant	115	105	105, 115
*Wmc805*	SSR, co-dominant	120	118	118, 120
*Gwm159*	SSR, co-dominant	350	332	332, 350
*SCAR112*	SCAR, dominant	115	-^a^	115
*SCAR203*	SCAR, dominant	205	210	205

*^a^No PCR products were obtained.*

### Allelism between *PmFG* and Previously Documented *Pm2* Alleles

Because *PmFG* was mapped on the chromosome arm 5DS, FG-1 was crossed with several powdery mildew resistant stocks with documented *Pm* genes on the same chromosome arm to determine the allelic relationship between *PmFG* and previously documented *Pm* genes. These included Ulka/8*Cc with *Pm2a*, Liangxing 66 with *PmLX66* and TD114 with *Pm2a+Pm6*. *Bgt* isolate B03 which is avirulent on FG-1 and the other lines was used to inoculate the large F_2_ populations. The phenotyping reactions of those F_2_ populations are shown in **Table 3**. No susceptible plants were recovered from the F_2_ populations of FG-1 and the three documented resistant stocks with *Pm2* alleles including reciprocal crosses. Therefore, *PmFG* seems to be an allele of the *Pm2* locus.

**Table 3:** Phenotype reactions of the F_2_ populations from the cross between FG-1 and the wheat lines Ulka/8*Cc with *Pm2a*, Liangxing 66 with *PmLX66* and TD114 with *Pm2a+Pm6* when tested with *Bgt* isolate B03.

Crosses	Number of resistant plants	Number of susceptible plants
FG-1 × Ulka/8*Cc	18,028	0
Ulka/8 × Cc/FG-1	6,112	0
FG-1 × Liangxing 66	4,324	0
FG-1 × TD114	6,235	0

### Multi-race Comparisons of FG-1 and Lines with Reported Resistance Alleles at or Near the *Pm2* Locus

FG-1 showed a clearly different response spectrum to the *Bgt* isolates from those of other lines with possible *Pm2* alleles and *Pm48* (Supplementary Table S1). Compared to the lines with *Pm2a*, *Pm2b*, *Pm2c*, *PmLX66*, *PmW14*, *PmZ155*, *PmX3986-2*, *PmWFJ*, *MlBrock*, and *PmD57-5D* and *Pm48*, there were differences in response for 6, 5, 8, 5, 6, 7, 8, 12, 5, 4, 6, and 7 *Bgt* isolates, respectively (Supplementary Table S1; **Figure 1**). FG-1 also showed a different response pattern from the putative *Agropyron cristatum* derivative PB3558 that carried *PmPB3558* in the *Pm2* chromosome region for the response to *Bgt* isolate B07 and B30 (Supplementary Table S1). Therefore, *PmFG* was different from the other *Pm* genes located at or near the *Pm2* locus.

### Variation in the Closely Linked Marker Alleles between FG-1 and Genotypes with *Pm* Genes Located at or Near the *Pm2* Locus

**FIGURE 4:**
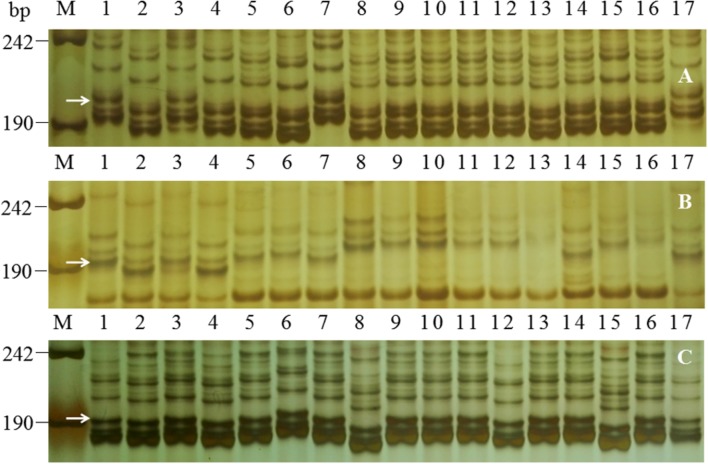
**Polymerase chain reaction (PCR) amplification patterns of FG-1 and wheat genotypes with documented *Pm* genes at or near the *Pm2* locus using SNP-derived markers *Bwm9***(A)**, *Bwm13***(B)**, and *Bwm16***(C)**.** M, DNA marker pUC18 *Msp* I; lanes 1 and 2, FG-1 and Mingxian 169; lanes 3 and 4, resistant and susceptible pools from FG-1 × Mingxian 169; lanes 5–17, genotypes with sequential order of UlKa/8*Cc (*Pm2a*), KM2939 (*Pm2b*), Niaomai (*Pm2c*), Tabasco (*Pm48*), Liangxiang 66 (*PmLX66*), Wennong 14 (*PmW14*), Zhongmai 155 (*PmZ155*), YingBo 700 (*PmYB*), X3986-2 (*PmX3986-2*), Brock (*MlBrock*) and D57-5D (*PmD57-5D*), Wanfengjian 34 (*PmWFJ*) and PB3558 (*PmPB3558*); white arrows indicate 208 **(A)**, 194 **(B)**, and 192 **(C)** bp polymorphic bands in FG-1.

To further compare *PmFG* and documented *Pm* alleles in the multi-allelic *Pm2* region, several markers associated with *PmFG*, including all the SNP-derived markers and the SSR marker *Cfd81*, were compared for their allelic variations among FG-1, Ulka/8*Cc, KM2939, Niaomai, Tabasco, Liangxing 66, Wennong 14, Zhongmai 155, YingBo700, X3986-2, Wanfengjian 34, Brock, D57-5D and PB3558 that carried *Pm* genes located at or near the *Pm2* locus. The *Cfd81*, *Bwm20* and *Bwm25* amplicons were approximately 270, 195, and 208 bp, respectively, in FG-1 and all lines with *Pm* genes at or near the *Pm2* locus. However, the presence of eight of 10 SNP-derived markers linked to *PmFG* (including *Bwm3*, *Bwm6*, *Bwm8*, *Bwm9*, *Bwm11*, *Bwm13*, *Bwm16*, and *Bwm21*) varied in their allelic sizes among FG-1 and various stocks with *Pm* genes at or near the *Pm2* locus (**Table 4**; **Figure 4**). For example, the two *Bwm21* amplicons in FG-1 were different from those in Ulka/8*Cc (*Pm2a*), Tobasco (*Pm48*) and Brock (*MlBrock*); and the 194 bp *Bwm13* amplicon was unique to FG-1 (**Table 4**). Therefore, FG-1 showed a diversity of alleles of the *PmFG*-linked markers compared to other resistant stocks with *Pm* genes at or near the *Pm2* locus. From our results of allelism tests, allelic variation of the linked markers and response spectrum analysis, we find that *PmFG* is a new allele of *Pm2*.

**Table 4:** Sizes of the linked marker alleles of FG-1 and the wheat genotypes with documented *Pm* genes at or near *Pm2* locus on chromosome arm 5DS using SNP-derived markers of *PmFG*.

Genotypes	Gene	*Bwm3*	*Bwm6*	*Bwm8*	*Bwm9*	*Bwm11*	*Bwm13*	*Bwm16*	*Bwm21*
FG-1	*PmFG*	144	169	210	208	225	194	192	220, 235
UlKa/8*Cc	*Pm2a*	145	160	194	199	225	196	192	210, 220, 225, 235
KM2939	*Pm2b*	148	160	190	197	225	197	195	220, 235
Niaomai	*Pm2c*	144	160	210	208	226	196	192	220, 235
Tabasco	*Pm48*	144	167	194	197	226	200	187	210, 220, 225, 235
Liangxing 66	*PmLX66*	145	165	196	199	228	201	192	220, 235
Wennong 14	*PmW14*	148	162	196	199	228	200	192	220, 235
Zhongmai 155	*PmZ155*	144, 145	165	196	199	228	200	192	220, 235
YingBo 700	*PmYB*	145	162	196	199	228	200	190	220, 235
X3986-2	*PmX3986-2*	144	169	194	197	225	204	192	220, 235
Brock	*MlBrock*	144	160	196	199	225	200	192	210, 220, 225, 235
D57-5D	*PmD57-5D*	144	162	196	199	226	200	187	220, 235
Wanfengjian 34	*PmWFJ*	148	162	196	199	228	201	190	220, 235
PB3558	*PmPB3558*	146	169	210	208	225	200	190	220, 235

*All the allelic sizes in this table are listed in bp.*

### Potential of Closely Linked Markers for MAS

**FIGURE 5:**
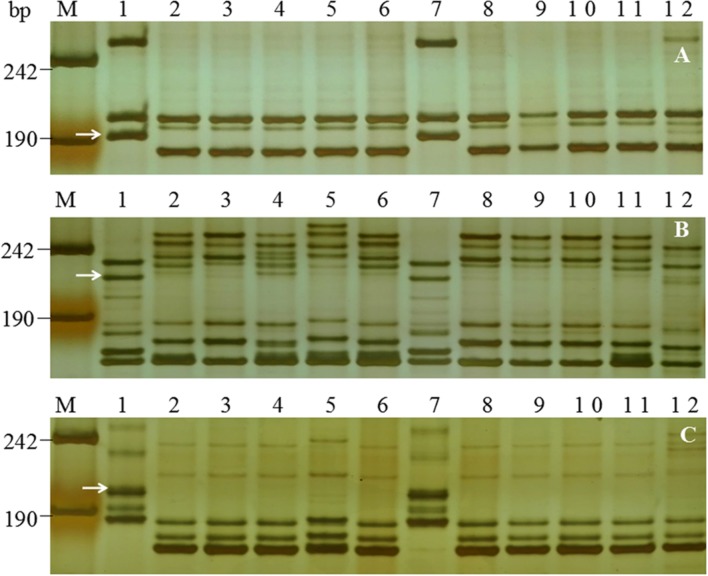
**Polymerase chain reaction amplification patterns of *PmFG*-linked SNP-derived markers *Bwm20***(A)**, *Bwm21***(B)**, and *Bwm25***(C)** in FG-1, Mingxian 169 and several wheat cultivars.** M, DNA marker pUC18 *Msp* I; lanes 1 and 2, FG-1 and Mingxian 169; lanes 3–12, wheat cultivars with sequential order of Yumai 13, Lumai 1, Xiaoyan 6, Jimai 19, Jimai 22, Yangmai 158, Yumai 18, Zhengmai 9023, Yumai 34, and Lumai 14. The white arrows indicate the 195, 220, and 208 bp polymorphic bands in FG-1.

To investigate the usefulness of the markers linked to *PmFG* in MAS, eight closely linked markers flanking *PmFG* were assayed on 31 Chinese elite cultivars (**Table 5**; **Figure 5**). The *Bwm20*, *Bwm21*, *Bwm25*, *Cfd81*, *SCAR112*, and *SCAR203* alleles in all tested cultivars, except Jimai 22, were different from those in FG-1, demonstrating that the six markers could be used in MAS if *PmFG* was transferred to these cultivars by conventional hybridization. Other markers such as *Bwm13* and *Bwm16* also could be used, but only in certain cross combinations.

**Table 5:** Validation of *PmFG*-linked SSR marker *Cfd81*, SNP-derived markers *Bwm13*, *Bwm16*, *Bwm20*, *Bwm21* and *Bwm25*, and SCAR markers *SCAR112* and *SCAR203* on 31 Chinese wheat cultivars in MAS.

Cultivar	Province	*Cfd81*	*Bwm13*	*Bwm16*	*Bwm20*	*Bwm21*	*Bwm25*	*SCAR112*	*SCAR203*
Shi 4185	Hebei	+	+	-	+	+	+	+	+
Shimai 15	Hebei	+	+	+	+	+	+	+	+
Shiyou 17	Hebei	+	+	+	+	+	+	+	+
Shixin 633	Hebei	+	+	-	+	+	+	+	+
Shixin 733	Hebei	+	+	-	+	+	+	+	+
Ji 5265	Hebei	+	+	-	+	+	+	+	+
Henong 827	Hebei	+	+	-	+	+	+	+	+
Han 6172	Hebei	+	+	+	+	+	+	+	+
Han 7086	Hebei	+	+	-	+	+	+	+	+
Kenong 199	Hebei	+	+	-	+	+	+	+	+
Kenong 9204	Hebei	+	+	-	+	+	+	+	+
Jishi 02-1	Hebei	+	+	-	+	+	+	+	+
Yumai13	Henan	+	+	+	+	+	+	+	+
Zhengmai 9023	Henan	+	+	+	+	+	+	+	+
Yumai 34	Henan	+	+	-	+	+	+	+	+
Yumai 2	Henan	+	+	-	+	+	+	+	+
Yumai 18	Henan	+	+	+	+	+	+	+	+
Aikang 58	Henan	+	-	-	+	+	+	+	+
Luomai 21	Shandong	+	+	-	+	+	+	+	+
Zhengmai 366	Shandong	+	+	-	+	+	+	+	+
Zhengyumai 9989	Shandong	+	-	-	+	+	+	+	+
Jimai 22	Shandong	-	-	-	-	-	-	-	-
Lumai 1	Shandong	+	+	-	+	+	+	+	+
Lumai 21	Shandong	+	+	+	+	+	+	+	+
Lumai 14	Shandong	+	+	+	+	+	+	+	+
Shannong 21	Shandong	+	+	+	+	+	+	+	+
Luyuan 502	Shandong	+	+	+	+	+	+	+	+
Yannong 19	Shandong	+	+	-	+	+	+	+	+
Xinong 6028	Shannxi	+	+	+	+	+	+	+	+
Xiaoyan 6	Shannxi	+	+	-	+	+	+	+	+
Yangmai 158	Jiangsu	+	+	-	+	+	+	+	+

*‘+’amplified marker allele differs from that in FG-1 implying that the marker might be useful for MAS, and ‘-’marker is similar to that in FG-1.*

## Discussion

FG-1 was a common wheat line introduced from France and provided by the germplasm bank of Shijiazhuang Academy of Agricultural and Forestry Sciences. It was resistant to many *Bgt* isolates originating from different wheat-producing regions in China (Supplementary Table S1). Compared with the currently deployed cultivars in China, FG-1 is resistant to several highly virulent *Bgt* isolates that defeated several of the popular wheat cultivars that are currently grown in China. This suggests that FG-1 is a valuable resistant germplasm, which could be used to complement the resistant genes currently deployed in cultivars in China. However, FG-1 was not resistant to all the races present in certain regions of China. Therefore, the gene in FG-1 needs to be combined with other effective resistance genes to increase the durability of resistance.

Genetic analysis demonstrated that a single dominant gene, designated *PmFG*, conferred resistance to powdery mildew in FG-1 at the seedling stage. Unlike in previous studies (e.g., Gao et al., 2012; Huang et al., 2012; Xue et al., 2012; Xiao et al., 2013; Lu et al., 2015), *PmFG* was investigated for its resistance to all the avirulent races tested in this study. Based on this information, the single dominant gene in FG-1 was more thoroughly shown to confer powdery mildew resistance to all the avirulent races. Using molecular markers, *PmFG* was mapped in the *Pm2* region on the short arm of chromosome 5D. Its allelic relationship with *Pm2* was confirmed by allelism tests.

Compared with previous linkage maps of the *Pm2* alleles, more markers were added to the *PmFG* linkage map particularly the SNP-derived markers, which increased the density of the linked markers at this locus. Many alleles have been identified in the *Pm2* chromosome region, such as *Pm2a* in the wheat landrace Ulka from the former Soviet Union (Pugsley and Carter, 1953; Briggle, 1969; McIntosh and Baker, 1970), *Pm2b* and *PmPB3558* from the putative *Agropyron cristatum*-derived breeding lines KM2939 and PB3558, respectively (Lu et al., 2015; Ma et al., 2015a), *Pm2c* from the Chinese landrace Niaomai (Xu et al., 2015), *PmX3986-2*, *PmWFJ*, and *PmD57-5D* from the common wheat lines X3986-2, Wanfengjian 34 and D57-5D in China, respectively (Ma et al., 2011, 2014, 2015b) and *PmLX66*, *PmZ155*, *PmW14*, and *PmYB* from Chinese wheat cultivars Liangxing 66 (Huang et al., 2012), Zhongmai 155 (Sun et al., 2015), Wennong 14 (Song et al., 2014), and YingBo700 (Ma et al., 2015c), respectively. Another closely linked gene *Pm48* is present in the cv. Tabasco (Gao et al., 2012).

In this study, *PmFG* was distinguished from these documented genes by its response spectrum and the allelic variation of the linked marker alleles of the SNP-derived markers. Previous studies indicated that the documented *Pm2* alleles shared several similar markers, and the allelic variation of the linked markers could not be detected. However, the advent of the next generation in sequencing technologies significantly reduces sequencing costs, making SNP markers increasingly important due to their abundance in the genome and their very simple genetic mode of inheritance (bi-allelic). Therefore, to further distinguish *PmFG* from the *Pm* genes at or near the *Pm2* locus, SNP-derived markers of *PmFG* were screened to distinguish the allelic sizes. For the first time, *Pm* genes at or near *Pm2* locus were studied for their allelic variation using SNP-derived markers. This will contribute to differentiate the variation in the *Pm2* locus.

Multiple allelism in disease resistance genes is not uncommon. In the case of powdery mildew resistance in wheat, multiple resistance alleles have been identified at *Pm1*, *Pm2*, *Pm3*, *Pm4*, *Pm5*, and *Pm24* (McIntosh et al., 2013). So far, 17 functional alleles have been identified at the *Pm3* locus, making it one of the largest allelic series of plant resistance genes (Bhullar et al., 2010). For other wheat disease resistance, multi-allelic loci have also been identified. For example, using physical mapping, mutation and complementation, the stem rust resistance gene *Sr50* locus revealed extensive diversity, and holds promise for the mining other effective resistance alleles (Mago et al., 2015). These types of genetic diversity may contribute to the genetic improvement of crops and detection of variation in the pathogen and host–pathogen interactions (Prada, 2009; Wicker et al., 2013). In this study, the gene *PmFG* was identified as a new allele located at the *Pm2* allelic cluster. Like the *Pm3* allele cluster, more and more *Pm2* alleles with different response spectra to *Bgt* isolates and allelic variation have been identified, increasing the diversity at this locus. However, to further distinguish these alleles, more research should be undertaken in the future, such as fine mapping of all the *Pm2* alleles, re-sequencing of the *Pm2* region, and even development of functional markers based on the cloning of functional genes of these alleles.

In order to transfer *PmFG* into the susceptible cultivars or to pyramid multiple R-genes effectively, MAS should be a high-priority in wheat breeding programs. In the previous studies, the SSR marker *Cfd81* was shown to be an effective marker for differentiating several *Pm2* alleles in MAS (Ma et al., 2015a,b,c). However, *Cfd81* serves as only a one-sided marker of *Pm2* alleles, and other markers like *SCAR112* and *SCAR203* also have limited roles because of their dominant characters, which do not allow homozygous and heterozygous genotypes to be distinguished (Ma et al., 2015a,b,c). More closely linked markers need to be screened to increase the density of applicable molecular markers for breeding. SNP markers are based on the variation of a specific nucleotide at a given sequence position between individuals, and therefore their numbers in the wheat genome should be much higher and their detection can be facilitated by cost-efficient based on chips or other array techniques (Colasuonno et al., 2014; Wang et al., 2014, 2015a). In this study, five co-dominant SNP markers were evaluated for their applicability in MAS. The SNP-derived markers *Bwm20*, *Bwm21*, and *Bwm25* flanked *PmFG* at genetic distances of only 0.3, 0.3, and 0.5 cM respectively, and they were diagnostic in 96.8% of the tested cultivars in this study. Therefore, these SNP-markers can be used effectively in wheat breeding in the future.

One interesting observation was that the marker alleles of FG-1 by the *PmFG*-linked markers were all same as those of the cultivar Jimai 22, although *PmFG* is located on a significantly different genetic locus from that of the powdery mildew resistance gene in Jimai 22, which was mapped on chromosome arm 2BL (Yin et al., 2009). Therefore, *PmFG* cannot be distinguished in Jimai 22 genomic backgrounds by the *PmFG*-linked markers. This may ascribe to the insufficient marker density at this locus. Future fining mapping and characterization of the haplotype of this locus may contribute to clarify this issue.

## Author Contributions

PM: experimental implementation, data analysis, and manuscript preparation. HX: production of the mapping population and the genetic map. LL: data analysis. HZ: experimental implementation. GH: MAS analysis. YX: production of the genetic map. XZ: germplasm creation and field investigation. DA: study concept and design.

## Conflict of Interest Statement

The authors declare that the research was conducted in the absence of any commercial or financial relationships that could be construed as a potential conflict of interest.

## Footnotes

1 http://cb.natesc.gov.cn
